# Resistance Inducers for the Protection of Pedunculate Oak (*Quercus robur* L.) Seedlings against Powdery Mildew *Erysiphe alphitoides*

**DOI:** 10.3390/plants12030635

**Published:** 2023-02-01

**Authors:** Krzysztof Turczański, Marta Bełka, Maciej Spychalski, Rafal Kukawka, Raghavendra Prasad, Marcin Smiglak

**Affiliations:** 1 Department of Botany and Forest Habitats, Faculty of Forestry and Wood Technology, Poznań University of Life Sciences, Wojska Polskiego 71d, 60-625 Poznań, Poland; 2 Poznan Science and Technology Park, Rubież 46, 61-612 Poznań, Poland; 3 Department of Entomology and Pathology, Faculty of Forestry and Wood Technology, Poznań University of Life Sciences, Wojska Polskiego 71c, 60-625 Poznań, Poland; 4 Innosil Sp. z o.o., Rubież 46, 61-612 Poznań, Poland; 5 Environmental Horticulture, Royal Horticultural Society (RHS), Wisley GU23 6QB, UK

**Keywords:** powdery mildew, pedunculate oak, benzothiadiazole, ionic liquids, chitosan

## Abstract

Oak powdery mildew caused by *Erysiphe alphitoides* (Griffon and Maubl.; U. Braun & S. Takam.) is a common disease in European forests. One of the most susceptible species is the pedunculate oak (*Quercus robur* L.). Presently, a few methods are available to control powdery mildew, e.g., the use of fungicides (e.g., based on citric acid), antagonistic fungi or bacteria, chemical treatments (e.g., sulphur, potassium bicarbonate) or genetic resistance. In our study, we aimed to check the effects of using chitosan derivatives and novel active substances inducing the plants’ natural resistance: benzodiathiadiazole (both in neutral and salt form). 84 pedunculate oak seedlings were subjected to the experiment in three treatment variants (plus positive and negative controls). The plants were treated with active substances and inoculated with *E. alphitoides*. Although the powdery mildew symptoms appeared in all variants, they were manifested mainly by the *mycelium* in the form of small spots. The experiment indicated that the highest limitation of powdery mildew *mycelium* was achieved by applying *N*-methyl-*N*-methoxyamide-7-carboxybenzo(1,2,3)thiadiazole (BTHWA). The application of BTHWA reduced disease development by 88.9% when compared to the effects of the other variants.

## 1. Introduction

Oak powdery mildew (PM) caused by *Erysiphe alphitoides* (Griffon & Maubl.) U. Braun and S. Takam., an exotic pathogen first described in Europe in 1907 [1], is one of the most common diseases of pedunculate oak (*Quercus robur* L.) and sessile oak (*Q. petraea* (Matt.) Liebl.) [2,3]. The disease can be very severe, particularly in young plants resulting in growth reduction and seedling losses [4,5,6,7,8]. The leaves affected by *E. alphitoides* suffer from decreased net photosynthesis [9,10] and changes in assimilation management that can affect tree growth and survival rate [11]. Even though PM is a primary foliage disease, the knowledge of the defence responses of the tree to infection by this pathogen is limited.

Protection against oak PM is implemented primarily in forest nurseries. So far, a few methods are available to control the disease, including some conventional fungicides, e.g., based on citric acid, antagonistic bacteria, e.g., *Streptomyces* [12] or fungi, e.g., *Trichoderma asperellum* IZR D-11 [13], commercial chemical treatments, e.g., with sulphur, potassium bicarbonate, potassium phosphite, salicylic acid [14] or by developing genetic resistance.

Systemic acquired resistance (SAR) of plants is a form of induced resistance that is activated throughout a plant after exposure to pathogens or chemical substances such as nicotinic acid, salicylic acid, benzothiadiazole or chitosan [15,16]. Induction of SAR is characterized by the accumulation of salicylic acid to stimulate defence mechanisms, often resulting in a localized hypersensitive response [17].

The signalling agents contributing to SAR are salicylic acid (SA) and several components of the SA pathway, including the methylated derivative of SA (methyl SA, MeSA) [18,19]. The precise mode of SA action is not yet described. It has become increasingly evident that SA is part of a complex network associated with plant response [20]. Treatment of plants with salicylic acid and functional analogues results in a plant response with increased expression of PR family genes, including NPR1. PR genes code for proteins such as β-1,3-glucanases and chitinases and play a role in either preventing or slowing the colonization of pathogens in the host [21].

One of the most studied active substances capable of inducing plant resistance is benzo(1,2,3)thiadiazole-7-carboxylic acid S-methyl ester (ASM, BTH), which is commercially available as Bion 50 WG and Actigard 50 WG marketed by Syngenta [22,23,24]. 

Our earlier studies were aimed at obtaining new derivatives of benzothiadiazoles that could act as effective plant resistance inducers [25,26,27,28,29,30]. Moreover, a group of investigated new derivatives showed lower (eco)toxicity and higher biodegradability than the commercially available BTH [31].

A substance of natural origin that has also been reported to induce plant resistance is chitosan, which belongs to the group of polysaccharides [32]. Chitosan is insoluble in most solvents, but it is possible to derivatize it using e.g., acetic acid, formic acid, succinic acid, lactic acid, and malic acid to obtain the salt form of chitosan which improves its solubility [33]. Chitosan has also shown antimicrobial activity against different groups of microorganisms, such as bacteria and fungi, including the fungi causing PM [34]. However, its antimicrobial action is affected by intrinsic factors such as the type of chitosan, degree of chitosan polymerization, the type of host, natural nutrient constituency, chemical or nutrient composition of the substrates or both, and the environmental conditions [35].

Induction of plant resistance has a positive effect on general plant health. A reliable plant health assessment might be performed using the Vegetation indices (VIs) [36,37]. VIs are a type of mathematical transformation of spectral bands characterizing the spectral properties of plants. The expressed values of different VIs, including the Normalized Difference Vegetation Index (NDVI) and Simple Ration Index (SR), help to analyze crop vigour and several other vegetation properties, including biomass and chlorophyll content [38]. Vegetation indices have also been shown to be reliable indicators of abiotic stress in plants [39,40].

Among the tested compounds, *N*-methyl-*N*-methoxyamide-7-carboxybenzo(1,2,3)thiadiazole (BTHWA), which is a derivative of benzothiadiazole in a neutral form, has shown the highest effectiveness of SAR induction [41]. The second substance with similar activity is a benzothiadiazole derivative in ionic form, cholinium benzo(1,2,3) thiadiazole-7-carboxylate ([CHOL][BTHCOO]). Both substances have been subjected to molecular biology studies to verify their action as SAR inducers [41]. After the treatment, the plants treated either with BTHWA or [CHOL][BTHCOO] solution showed a higher level of SAR marker genes NPR1, PAL, and PR-1b at 4 h than the untreated control. Moreover, 7 days after infection of plants treated with both above-mentioned compounds, the level of viral RNA accumulation was lower compared to this of untreated control plants, which leads to the conclusion that the replication of the virus was less efficient. These experiments showed that both BTHWA and [CHOL][BTHCOO] act only through plant metabolism and do not possess antifungal properties [41].

The general aim of the study was to check the effect of using novel substances representing benzothiadiazoles, a BTH derivative, namely: *N*-methyl-*N*-methoxyamide-7-carboxybenzo(1,2,3)thiadiazole (BTHWA); cholinium benzo(1,2,3)thiadiazole-7-carboxylate ([CHOL][BTHCOO]) and chitosan lactate (CHL) being SAR elicitor of natural origin, on the protection of pedunculate oak against the PM. We hypothesized that the above-mentioned substances would strengthen the oak’s natural defence mechanisms in response to fungal inoculation and, consequently, trigger the oak’s response against the PM.

## 2. Results

**Figure 1:**
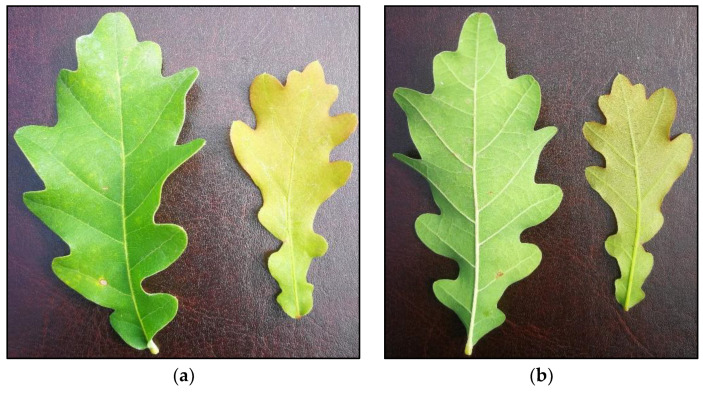
Oak leaves (mature—Left; juvenile—Right) with no symptoms of powdery mildew after 69 days of fungal inoculation and five treatments with BTHWA: (**a**) upper side; (**b**) bottom side.

**Figure 2:**
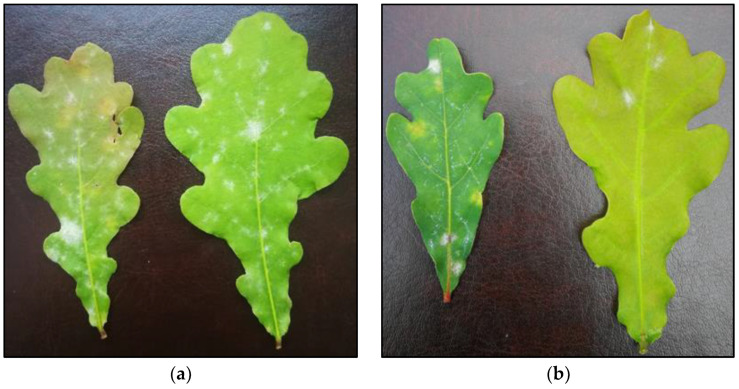
Powdery mildew *mycelium* in the form of small spots in oak leaves after 69 days of fungal inoculation and five treatments with (**a**) [CHOL][BTHCOO]; (**b**) BTHWA.

**Figure 3:**
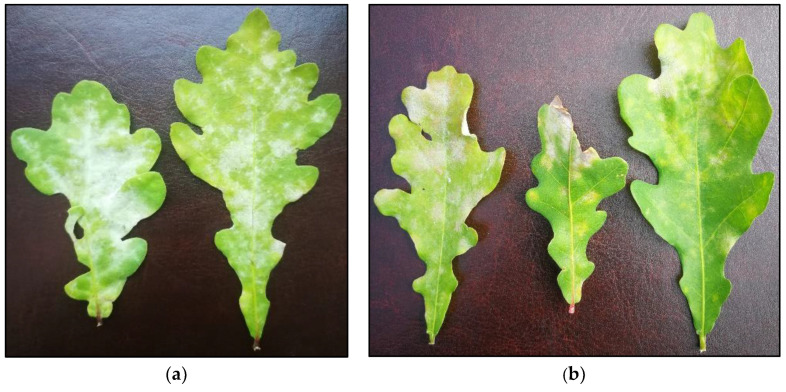
Powdery mildew *mycelium* scattered over a larger area after 69 days of fungal inoculation: (**a**) C+; (**b**) after five treatments with CHL.

Plants subjected to the study were treated with the test substances five times in total, including two treatments before fungal inoculation, as described in Table 1. After the inoculation, the number of PM symptoms started to appear and increase in time. The first visible symptoms of PM, mostly manifested by small *mycelium* patches up to 7 mm in diameter, appeared seven days after the inoculation. Interestingly, they were present only on the plants subjected to both controls and were located mostly on top leaves. On the 35th day after the fungal inoculation, the symptoms appeared in all treatments (Table 1) and started to vary. *Mycelium* was limited to small spots in the seedlings treated by [CHOL][BTHCOO] and BTHWA. On the plants subjected to other treatments, a higher presence of more scattered (diffuse) *mycelium* of size over 7 mm was observed (Figure 1, Figure 2 and Figure 3). Furthermore, severe tissue necrosis and leaf defoliation were not observed.

Among the groups of seedlings treated with the tested substances (all variants), the highest number of plants with symptoms on the 49th and 69th day after inoculation was observed among those treated with CHL. Interestingly, the percentage of seedlings with symptoms on the second and third dates of assessments was lower when compared to those treated with [CHOL][BTHCOO]. However, the development of the disease accelerated considerably in the subsequent days. The substance producing the greatest reduction in the number of plants with disease symptoms was BTHWA. Its application resulted in 11% of seedlings with symptoms, compared to 73% of plants with symptoms among the seedlings of C+ and 56%—for plants of C− (Table 1). An interesting but not surprising result was a high infection of plants in C−. It shows that the pathogen was present in the environment and, therefore, naturally infected the uninoculated plants in the experiment (See also DSI index; Table 2).

On the fourth measurement date, the PM infection of leaves was evaluated in more detail. A similar evaluation was repeated on the 69th day after inoculation with the fungus to determine whether the disease was still developing. The results are expressed in terms of the DSI index in Table 2. The most effective protection was provided by the BTHWA (DSI index−3%). Furthermore, it should be emphasized that only on the plants treated with this substance no further development of the pathogen was observed. The second lowest value of DSI index was recorded for the plants treated with [CHOL][BTHCOO]. The seedlings treated with CHL, despite initially good values in terms of the number of infected plants (Table 1), showed the poorest treatment effects at the end of the experiment. Moreover, the assessment made on the 69th day provided practically the same result as that of the C−.

Additionally, the plants subjected to all treatments were analysed in terms of their growth (Table 3). As indicated before, infection with PM might lead to a reduction in biomass increase. Although no statistically significant differences were observed between the plants representing all treatments, the plants sprayed with the tested substances showed a higher growth rate than the plants from inoculated control variant.

In the presented study, no significant differences were observed between VIs measured for plants before and after treatment and inoculation (Table 4). The NDVI values for the plants subjected to different treatments before infection were in the range of 0.71–0.73, while after infection, they were in the range of 0.67–0.73. The SR values ranged from 5.94 to 6.70 and 5.15 to 6.37 for the plants subjected to all treatment variants before and after infection, respectively. 

## 3. Discussion

There is a plethora of reports on the investigation of plant defence mechanisms through studies on herbaceous annuals or short-lived perennials which include model plant species such as *Arabidopsis thaliana* (mouse-ear cress), *Cucumis sativus* (cucumber), and *Nicotiana tabacum* (tobacco). However, not much attention has been paid to investigating these phenomena for trees, both angiosperms and gymnosperms [42].

Trees have some unique features when compared with herbaceous plants: they are usually much larger, have much longer life spans (sometimes of millennia), are characterized by life histories that have no equals among herbaceous model plants, and exhibit different architectural forms linked to secondary growth. Trees may be subject to different patterns of herbivore and pathogen pressure and require different protection modes. Hence, findings from reports on herbaceous studies might not always apply to forest trees [43].

The activity of benzothiadiazoles related to the induction of plant resistance against PM has been reported on various crops such as cucumber [44], strawberry [45] or wheat [46]. In addition, the activity of the BTHWA substance was confirmed in our previous studies performed on ash seedlings (*Fraxinus excelsior* L.). After the application of this substance, the development of the fungus *Hymenoscyphus fraxineus* was significantly slowed down or even completely stopped [47]. Moreover, the treatment with BTHWA limited the size of necrotic phloem lesions [47]. According to preliminary unpublished data, BTHWA and [CHOL][BTHCOO] have no direct effect on the fungi species such as *Alternaria alternate*, *Trichoderma viride* and *Oidium neolycopersici* causing PM on tomato.

In the case of PM, we decided to check the efficiency of the tested substances in reducing disease symptoms. It is crucial, especially considering that *E. alphitoides* affects oaks of all ages, but primarily young plants, both in the renewals and forest nurseries, by reducing their growth and causing seedling losses [5,6,7,8]. Our study showed that the first visible symptoms of PM, mostly manifested by small *mycelium*, appeared seven days after inoculation in both controls. During the experiment, the symptoms occurred in the plants subjected to the other variants of treatment. However, the appearance of PM in uninoculated control was interesting but not surprising. It shows that the PM spores were present in the open air and could naturally infect seedlings stored in the open area. Moreover, this indicates that both artificially and “naturally” infected controls resulted in a higher number of seedlings with PM symptoms, i.e., 73% (C+) and 56% (C−), than plants sprayed with BTHWA and [CHOL][BTHCOO].

Comparing the plants with the induced resistance against PM, the lowest number of symptoms was noted in the plants treated with BTHWA (11%) and [CHOL][BTHCOO] (31%). In these treatments, we observed that the *mycelium* was limited only to small spots, whilst they were more scattered in the controls and plants treated with CHL. These observations are supported by the values of DSI index which were the lowest for the plants treated with BTHWA (3%). Moreover, the DSI index for the seedlings treated with BTHWA took equally low values after the 49th and 69th days after inoculation. In other words, we can assume that the natural defence mechanisms triggered by the applied substance were still active.

As for the results obtained for the plants treated with CHL, we assume that the initially low percentage of seedlings showing symptoms of PM resulted from CHL showing both plant resistance-inducing activity and antifungal properties. The observations made on the 21st and 35th day after inoculation suggest that such a double action could provide better protection than the chemical inducer [CHOL][BTHCOO]. However, during the experiment, it turned out that the protection provided by CHL was broken, and the development of the pathogen significantly accelerated. Our results are in line with literature data describing chitosan as plant resistance inducers with lower efficacy than benzothiadiazoles [48].

During the experiment, we also observed that the seedlings sprayed with the tested substances showed higher mean growth than plants subjected to untreated control. Most likely, this resulted from a lower degree of PM infection, which is known to have a significant effect on the growth of young trees [4,5,6,7,8].

It has been reported in the literature that NDVI is a reliable indicator of plant health [36,37] and VIs are sensitive to abiotic stress responses in plants [39,40]. Thus, we checked the plant’s response to PM using selected spectral indices, i.e., NDVI and SR. However, the results showed no statistically significant differences (Table 4). The NDVI values indicated that before and after inoculation, the tested treatment variants maintained plant health, and the applied substances did not induce abiotic stress on plants. We assume that the lack of VIs response may be connected with the slight PM symptoms, i.e., without severe defoliation or leaf necrosis observed in all treatments. Additionally, both NDVI and SR values after inoculation in C− group tended to decrease slightly. In order to substantiate this, more frequent measurements are needed. Moreover, more sensitive approaches, including Chlorophyll fluorescence [49] and gas exchange measurements, can be considered for future studies.

This preliminary study showed that the tested substances, especially BTHWA, have a high potential among resistance inducers to protect oak seedlings against the PM. However, we still need to conduct more research to elucidate the protection’s mechanisms in more detail. Perhaps the modification of the measurement methodology and its extension to include other parameters, i.e., related to the fast kinetics of chlorophyll, will allow a better understanding of the plant’s response. Moreover, in the future study, we plan to analyse physiological features that can be affected by PM [50,51,52].

Moreover, to prove that such a plant response was caused by SAR induction, it would be necessary to supplement these results with detailed biochemical studies on enzymes or molecular studies on genes characteristic of this phenomenon. For instance, monitoring of changes in the level of expression of SAR marker genes in subsequent days after treatment will allow for a more accurate demonstration of how long the plant remains in a stimulated state. In addition, such a comparative analysis performed for the tested substances will allow for a more accurate comparison of their effectiveness on a given experimental model.

Detailed analyses may also concern a more accurate estimation of the level of infection, which can be performed several times after infection. This will allow for monitoring of the level of disease development. Such a study should also be supplemented with histo-cytochemical analysis of leaf symptoms, as their source might be an abiotic factor and not a pathogen attack [53]. Similar studies on plant response to treatment with different concentrations of BTH have already been conducted and the presented methodology can be applied to our further tests [54,55].

Nevertheless, our study is a “first step” in this area, and we believe that the testing of the activity of new substances capable of providing effective protection to trees will be of key importance for forestry.

Considering the results obtained, we conclude that among administered substances used to strengthen the oak’s natural defence mechanisms in response to *E. alphitoides* inoculation, the highest efficiency in reducing the disease development had BTHWA. The study is the first report of using benzothiadiazoles to protect oak against PM.

## 4. Materials and Methods

### 4.1. Material Collection

In early June 2022, we collected 100 two-year-old oak seedlings (*Q. robur*) from a forest nursery in the Lipka Forest District, NW Poland. However, we have used only 84 plants of approximately 10–15 cm in height. The rest of seedlings were removed as we wanted to have a similar starting height point. The seedlings were collected together with soil and placed directly into pots with a capacity of four litres. The plants were stored in an open place and watered once or twice a week to keep the soil moist. All collected oaks showed no visible symptoms of PM or other diseases. The inspection was repeated twice, i.e., during the collecting of the plants from the forest nursery and on the same day when the fungal inoculation was performed.

### 4.2. Tested Substances

The substances studied were two benzothiadiazole derivatives obtained in our group: *N*-methoxy-*N*-methylbenzo(1,2,3)thiadiazole-7-carboxamide (BTHWA) [56,57], cholinium benzo(1,2,3)thiadiazole-7-carboxylate ([CHOL][BTHCOO]) [30], and a Chitosan lactate (CHL). BTHWA, [CHOL][BTHCOO] and CHL were provided by company Innosil Ltd. (Poznan, Poland). The tested substances were dissolved in water in the following concentrations: BTHWA—20 mg/L, [CHOL][BTHCOO]—25 mg/L, and CHL—400 mg/L.

### 4.3. Experimental Design

Two weeks after putting the seedlings into pots, we applied the tested substances for the first time. The experiment included five variants of treatment (Table 5). The tested substances were applied to plants by foliar application every 14 days. Each plant was sprayed with 10 mL of a working solution containing a specified concentration of the tested substance. The plants were sprayed in the evenings. After two treatments, the seedlings, except those representing C−, were inoculated with fungus (seven days after the second treatment). Subsequently, the third application of the tested substances was made after the next seven days. Altogether, five applications were performed between the end of June and August 2022.

### 4.4. Seedling Inoculation

Inoculation was performed on the 7th day after the second application of the tested substances. To obtain sufficient inoculum of the *E. alphitoides* isolates, 30 min before inoculation, fresh leaves with visible symptoms of PM were collected from an infected *Q. robur* (52°42′6051″ N, 16°90′0498″ E). Subsequently, the leaves were placed in paper bags and transported to the experiment site. The seedlings were inoculated late in the evening to allow the fungi to germinate in the morning dew and to protect them from the hot air during the day. The leaves were inoculated by gently rubbing the adaxial epidermis of three expanded leaves with sporulating colonies on the surface of the source leaves. Inoculation was performed once.

### 4.5. Disease Severity Assessment 

The pathogen infection with visible symptoms on leaves was assessed every 14 days, on the same dates as the five treatments, before the tested substances were applied. Moreover, two additional observations were made when the application of substances was stopped (on the 49th and 69th day). The percentage of seedlings with visible symptoms of *mycelium* on the plants subjected to each variant was determined to check the progress of the disease development. More detailed analyses were performed when the application of substances was stopped, i.e., on the 49th and 69th day after the fungus inoculation. The plants were rated for infection on a scale of 0–4: 0, no infection; 1, less than 10% leaves affected by *mycelium*; 2, 11–25% leaves affected; 3, 26–50% leaves affected; 4, more than 50% leaves affected. Plant pathologists, breeders, and professionals in other disciplines often simplify the ordinal scale rating data by transforming these numbers into a disease severity index (DSI) [58,59]. The DSI is a single index number for summarising the total effect of the disease on a single plant or a small sample of plants. The calculation was made according to the equation:

$$\mathrm{DSI}\;[\%]\;\frac{\sum^{\;}\left(class\;frequency\times score\;of\;rating\;class\right)}{\left(total\;number\;of\;observations\right)\times \left(maximal\;disease\;index\right)}\times 100$$

The simplification of disease severity resulted from the observed symptoms of PM in our experiment—mostly small *mycelium* in the form of spots up to 7 mm in diameter or less frequent—*mycelium* which scattered on a larger area of leaves (over 7 mm). Furthermore, we did not note severe leaf defoliation or tissue necrosis.

### 4.6. Vegetation Indices Collection

In the presented study, spectral vegetation indices such as NDVI and SR were measured (Table 4). The NDVI was calculated based on the equation NDVI = (R_NIR_ − R_RED_)/(R_NIR_ + R_RED_) [30], and the SR index was based on the equation SR = R_NIR_/R_RED_ [60,61]. Two repetitions were made during the experiment. The first measurement was carried out on the 10th day of the experiment (before treatment and inoculation) to access the initial spectral properties of the plants not treated either with active substances or infected with PM. The second measurement was carried out on the 69th day after fungal inoculation (after treatment and inoculation measurements). The selected and marked fully matured leaves (one leaf in an individual plant) were measured using the handheld device PolyPen RP 410 (Photon Systems Instruments Ltd., Drásov, Czech Republic). The leaf (held in place with a RP 410 mechanical leaf holder) was exposed to an internal light source of RP 410 (Xenon incandescent lamp with a spectral range of 380–1050 nm) with a UVIS sensor (380–790 nm). Five plants representing each variant of treatment were measured, including controls. The recorded data were downloaded and exported using integrated Spectrapen software from the device provider (Photon Systems Instruments Ltd., Drásov, Czech Republic).

### 4.7. Statistical Analysis

Morphological parameters of oak seedlings and VIs are means ± Standard Deviation (SD). Data were evaluated by analysis of variance (ANOVA), and the mean differences were compared by post hoc test at a *p* < 0.05 level, according to Tukey’s HSD. Statistical analyses were performed using OriginLab 2022 software for Windows (OriginLab Corp., Northampton, MA, USA). The Disease Severity Index (DSI%) and the data of seedlings with visible symptoms of PM are given as percentages per treatment. Such an approach comes from the visual assessment of symptoms based on the sum of visual scoring data but not mean values. Hence, no statistical analysis was performed for this scoring data set.

## Figures and Tables

**Table 1:** The percentage of seedlings with visible symptoms of powdery mildew on the following days after inoculation.

Treatment Variants	Days Post Fungal Inoculation				
	7th Day	21st Day	35th Day	49th Day	69th Day
C+	13.3%	60.0%	66.7%	73.3%	73.3%
CHL	-	5.9%	23.5%	52.9%	58.8%
[CHOL][BTHCOO]	-	18.8%	25.0%	31.3%	31.3%
BTHWA	-	-	5.6%	11.1%	11.1%
C−	5.6%	38.9%	50.0%	55.6%	55.6%

**Table 2:** The Disease Severity Index of seedlings on the 49th day and the 69th day after inoculation.

Treatment Variants	49th Day	69th Day
	DSI	DSI
C+	52%	53%
CHL	28%	34%
[CHOL][BTHCOO]	11%	13%
BTHWA	3%	3%
C−	32%	38%

Disease Severity Index (DSI%).

**Table 3:** Plants’ height at the beginning and end of the experiment.

Treatment Variants	Mean Initial Height (cm)	Mean Final Height (cm)	Mean Plant Growth (cm)
C+	12.9	13.8	0.9
CHL	12.2	15.7	3.5
[CHOL][BTHCOO]	13.3	15.8	2.4
BTHWA	12.6	14.3	1.7
Significance	ns *	ns	ns

ns * not significant at *p* < 0.05, according to Tukey’s HSD.

**Table 4:** Measured Vegetation indices (VIs) in the study.

Treatment Variants	NDVI		SR	
	Before Treatment and Inoculation	After Treatment and Inoculation	Before Treatment and Inoculation	After Treatment and Inoculation
C+	0.73 ± 0.02	0.71 ± 0.02	6.34 ± 0.36	5.94 ± 0.43
CHL	0.71 ± 0.03	0.70 ± 0.04	5.94 ± 0.74	5.63 ± 0.80
[CHOL][BTHCOO]	0.73 ± 0.02	0.74 ± 0.03	6.70 ± 0.65	6.37 ± 0.64
BTHWA	0.71 ± 0.02	0.72 ± 0.02	6.10 ± 0.47	6.04 ± 0.54
C−	0.71 ± 0.04	0.69 ± 0.02	6.09 ± 0.96	5.48 ± 0.37
Significance	ns *	ns	ns	ns

ns * not significant at *p* < 0.05 according to Tukey’s HSD (*n* = 5). Normalized Difference Vegetation Index (NDVI), Simple Ratio Index (SR).

**Table 5:** The variants of treatment in the experiment and simplified schedule of treatment.

Treatment Variants	N. of Plants	1 st VIs Measurement	Treatments Prior Inoculation (2 Times)	Fungus Inoculation	Treatments Post Inoculation (3 Times)	2nd VIs Measurement
C+	15	✓		✓		✓
CHL	17	✓	✓	✓	✓	✓
[CHOL][BTHCOO]	16	✓	✓	✓	✓	✓
BTHWA	18	✓	✓	✓	✓	✓
C−	18	✓				✓

Vegetation indices (VIs) measured: Normalized Difference Vegetation Index (NDVI), Simple Ratio Index (SR).

## Data Availability

The data presented in this study are available on request from the corresponding author.
